# Root anoxia effects on physiology and emissions of volatile organic compounds (VOC) under short- and long-term inundation of trees from Amazonian floodplains

**DOI:** 10.1186/2193-1801-1-9

**Published:** 2012-07-27

**Authors:** Araceli Bracho-Nunez, Nina Maria Knothe, Wallace R Costa, Liberato R Maria Astrid, Betina Kleiss, Stefanie Rottenberger, Maria Teresa Fernandez Piedade, Jürgen Kesselmeier

**Affiliations:** 1 Max Planck Institute for Chemistry, Hahn-Meitner-Weg 1, 55128 Mainz, Germany; 2 Instituto Nacional de Pesquisas da Amazônia, Av. André Araújo, 2936 Manaus, Brazil; 3 Universidade do Estado do Amazonas, Av. Djalma Batista, Chapada, 69050-010 Manaus, AM Brazil; 4 ORGENTEC Diagnostika GmbH, Carl-Zeiss-Str. 49-51, 55129 Mainz, Germany

**Keywords:** Short and long-term inundation, Waterlogging, Photosynthesis, Amazonian trees, Floodplains, Igapó, Várzea, Volatile Organic Compounds

## Abstract

Volatile organic compound (VOC) emissions are affected by a variety of biotic and abiotic factors such as light intensity, temperature, CO_2_ and drought. Another stress factor, usually overlooked but very important for the Amazon region, is flooding. We studied the exchange of VOCs in relation to CO_2_ exchange and transpiration of 8 common tree species from the Amazonian floodplain forest grown up from seeds using a dynamic enclosure system. Analysis of volatile organics was performed by PTR-MS fast online measurements. Our study confirmed emissions of ethanol and acetaldehyde at the beginning of root anoxia after inundation, especially in less anoxia adapted species such as *Vatairea guianensis*, but not for *Hevea spruceana* probably due to a better adapted metabolism. In contrast to short-term inundation, long-term flooding of the root system did not result in any emission of ethanol or/and acetaldehyde. Emission of other VOCs, such as isoprenoids, acetone, and methanol exhibited distinct behavior related to the origin (igapó or várzea type of floodplain) of the tree species. Also physiological activities exhibited different response patterns for trees from igapó or várzea. In general, isoprenoid emissions increased within the course of some days of short-term flooding. After a long period of waterlogging, VOC emissions decreased considerably, along with photosynthesis, transpiration and stomatal conductance. However, even under long-term testing conditions, two tree species did not show any significant decrease or increase in photosynthesis. In order to understand ecophysiological advantages of the different responses we need field investigations with adult tree species.

## Background

During the last decade, investigations within the framework of the Large-Scale Biosphere-Atmosphere Experiment (LBA) in Brazil have substantially increased our knowledge on biogenic VOC emissions from Amazonian tropical rainforest ecosystems (Kesselmeier *et al.*[2009]). However, these studies also demonstrated that we need to improve our knowledge in order to better understand inconsistencies between emissions, atmospheric concentrations and their contributions to secondary aerosol and cloud formation. These processes are governed by biogenic precursors and behave distinctly differently in the mostly pristine amazon region than in polluted regions, at least during the wet season (Pöschl *et al.*[2010]). Within this context, the differing environmental conditions during wet and dry seasons are of special interest. Central Amazonian floodplain areas are periodically flooded for several months per year (Junk [1989], Melack *et al.*[2010]). These flooded forests are characterized either as (i) várzea having nutrient-rich and sediment carrying whitewater, as (ii) igapó having brownish water containing only low quantities of nutrients and sediments and exhibiting a lower pH or as (iii) igapó clearwater areas with greenish water having an intermediate amount of nutrients and low amounts of humic substances (Prance [1979], Sioli [1954, 1956]). According to Junk *et al.* ([2011]) and papers cited therein, the total Amazonian floodplain area easily ranges up to 700,000 km^2^. As reported by Melack and Hess ([2010]) whitewater river floodplains alone contribute with more than 400,000 km^2^ whereas estimates for the igapó related to the Rio Negro add up to 118.000 km^2^ and further clearwater regions contribute with additionally 70,000 km^2^.

To survive the flooding period with its anoxic conditions for the root system, vegetation has developed several morphological, anatomical and physiological strategies (Parolin [2009], Parolin *et al.*[2004]). Many species react by leaf shedding and metabolic down regulation (Waldhoff and Furch [2002]), whereas other species maintain their leaves for several months even below water. Despite several morphological and anatomical strategies of the plant to provide oxygen to the roots, such as an increased number of lenticels, adventitious roots and aerenchyma (Parolin *et al.*[2004]), gas exchange as well as chlorophyll content can be affected by flooding (Waldhoff and Furch [1998, 2002]). Under waterlogged conditions, even adapted species reduce the assimilation rate in the aerial leaves, though in some specific cases an increase was reported (Parolin [2000a, 2001b], Parolin *et al.*[2004]).

Anaerobic dissimilation of carbohydrates in roots serves as an additional strategy employed by plants for surviving anoxic conditions. Alcoholic fermentation results in the production of ethanol, a toxic metabolite which is transported into the leaves by the transpiration stream. From there it can either be directly emitted into the atmosphere, or can be re-metabolized to acetaldehyde and/or acetate. Both of these compounds are still volatile enough to be partly released into the atmosphere (Kreuzwieser *et al.*[1999b], Rottenberger *et al.*[2008], Copolovici and Niinemets [2010]). Furthermore, fermentation within the leaf and exchange with the atmosphere may also contribute this emission in correlation to stomatal conductance and atmospheric compensation points (Winters *et al.*[2009]).

Hence, the release of these fermentation products may be of relevance for the pool of atmospheric oxygenated compounds. This is of special importance, as short-chain oxygenated compounds may play an important role in atmospheric chemistry (Karl *et al.*[2003], Kirstine *et al.*[1998], Lamanna and Goldstein [1999], Schade and Goldstein [2001, 2002], Seco *et al.*[2007]). These data are of special interest as substantial atmospheric amounts of these compounds are reported for Amazonia (Rizzo *et al.*[2010]). The fate of oxygenated volatiles is a matter of debate (Karl *et al.*[2010]). Furthermore, plant stress induced by flooding might also affect other VOC emissions such as isoprenoids. However, little is known about the anoxia induced release of volatiles from Amazonian tree species (Rottenberger *et al.*[2004, 2008]). Furthermore, these studies only report about short-term induction of a few days.

The present study was performed with seedlings collected in the two Amazonian environments várzea and igapó, the nutrient rich white water and nutrient poor black water floodplains, respectively (see above). Additionally, we used unpublished data sets on isoprenoid release carried out with the Amazonian tree species *Laetia corymbulosa* and *Salix martiana* in the year 2000 at the University of Oldenburg. For the corresponding emission of fermentation products see Rottenberger *et al.* ([2008]). The experiments were designed to investigate assimilation, transpiration, stomatal conductance, and VOC emissions affected by short-term and long-term flooding periods.. Furthermore, the results of the study on the emission of isoprenoids will be presented here for the first time for arboreal plants of floodplain forests and will be compared between species and ecotype as well as with the current information for tropical forests in general.

## Results

### Environmental conditions

#### *Micrometeorological conditions during the short-term flooding experiment*

Photosynthetic active radiation (PAR), air and leaf temperature, relative humidity and CO_2_ concentration inside the enclosure system were monitored before and during the flooding experiment during the three to four day inundation period (see Table 1**1**). Light intensities were successfully maintained constant at 498.4 ± 1.6 μmol m^-2^ s^-1^ by artificial radiation (see Section 5. Methods). Data of relative humidity (RH) and temperature (T_leaf_ and T_enclosure_) reflect quite constant conditions at the measurement site, showing values of 75–81%, 32–33 °C and 31.4 - 32.3 °C respectively (see Table 1). Almost no difference between T_enclosure_ and T_leaf_ was detected, probably due to the efficient mixing of the air in the enclosure, which allowed the leaf temperature to range around ambient temperature (Cook and Dixon [1964]). Carbon dioxide concentrations were 414 ± 21 ppm.

**Table 1:** **Environmental parameters during the short-term flooding experiments performed with*Vatairea guianensis*and*Hevea spruceana*under artificial illumination (LED, see Section 5)**

Plant Species	En	PAR [μmol m^-2^ s^-1^]	T_leaf_[°C]	T_enclosure_ [°C]	RH [%]	CO_2_ [ppm]
*Vatairea guianensis*	v	499 ± 5	33 ± 0.8	32.3 ± 0.6	77 ± 5	410 ± 7
	i	498 ± 4	32 ± 0.5	31.6 ± 0.8	81 ± 4	408 ± 9
*Hevea spruceana*	v	499 ± 1	32 ± 0.1	31.4 ± 0.1	78 ± 1.4	401 ± 6
	i	499 ± 1	32.7 ± 0.3	32.1 ± 0.3	75 ± 3	404 ± 2

Given are day time averages (5 minutes each) over the three to four day measurement period for two individuals of each tree species (n = 142 per day) ± SD. V and I indicate measurement cycles with tree species from the várzea (v) and igapó (i) environments (En), respectively.

In a previous experiment in 2000 (University of Oldenburg) greenhouse lighting conditions during the short-term flooding carried out with *L. corymbulosa* and *S. martiana* were maintained constant at 185 ± 36 and 216 ± 39 μmol m^-2^ s^-1^ for *L. corymbulosa* and *S. martiana,* respectively. Leaf temperature was very similar to that in our experiments and varied between 32.2 ± 3.3 and 32.7 ± 3.3 °C for *L. corymbulosa* and *S. martiana,* respectively. For more details of the micrometeorology and physiology of this experiment see (Rottenberger *et al.*[2008]).

#### Micrometeorological conditions during the long-term flooding experiment

For the long-term experiments we summarize the data for the time period before the start of inundation (nonflooded) and for the time at the end of the two months and three weeks incubation. Since the control of the light intensities was highly limited, natural conditions had to be taken into account. In order to differentiate possible influences of these highly variable light intensities on assimilation and VOC emissions, the data are sorted for different ranges of light intensities (see Table 2), demonstrating that assimilation and VOC emission values at the lower ranges of PAR are comparable to those measured at high PAR. These results were in accordance with prevailing measurements, monitoring all plants with the WALZ GFS300 photosynthesis instrument. Within these measurements we found photosynthesis to be light saturated around 300 μmol m^-2^ s^-1^ of photosynthetic active radiation (PAR; Data not shown) with all tree species investigated. Thus, light intensities under natural ambient conditions during both sets of experiments were variable, but were always sufficient to reach the maximum of the net photosynthesis rate. Therefore, PAR was not considered to be a limiting factor for photosynthesis and VOC emissions.

**Table 2:** **Micrometeorological parameters (Photosynthetic Active Radiation (PAR), leaf and enclosure Temperature, Relative Humidity, CO**_**2**_**concentrations), physiological parameters (CO2 Assimilation Rate (A), Transpiration (Tr), stomatal conductance (gs)) and Isoprenoids ((i) isoprene and (m) monoterpene) and oxygenated VOC (oVOC) ((me) methanol and (a) acetone) standard emission factors during the long-term flooding experiment**

Plant species	En	nf/f	n	PAR-Range	PAR [μmol m^-2^ s^-1^]	T_leaf_[°C]	T_enclosure_[°C]	RH [%]	CO_2_[ppm]	A [μmol*m^-2^*s^-1^]	Tr [mmol*m^-2^*s^-1^]	gs [mm s^-1^]	Isoprenoids [μg g^-1^ h^-1^]	oVOC [μg g^-1^ h^-1^]
*Garciniamacrophylla*	i	nf	52–89	1000–1735	1484 ± 147	37.1 ± 1.8	35.4 ± 0.9	33.3 ± 7.5	347.5 ± 4.0	1.8 ± 0.4	1.5 ± 0.2	1.0 ± 0.3	16.2 ± 3.0 (is)	1.6 ± 0.6 (me)
			4-8	800-1000	889 ± 74	35.2 ± 1.6	34.5 ± 1.5	35.9 ± 6.7	346.6 ± 6.7	1.9 ± 0.5	1.4 ± 0.3	1.1 ± 0.2	15.7 ± 5.7 (is)	2.2 ± 1.5 (me)
			10-21	300-800	471 ± 159	33.3 ± 3.0	32.7 ± 2.2	51.3 ± 16.1	351.5 ± 12	1.6 ± 0.8	1.2 ± 0.4	1.4 ± 0.5	13.3 ± 4.8 (is)	1.8 ± 0.3 (me)
		f	245-430	582-779	614 ± 33.6	32.2 ± 2.1	31.1 ± 2.1	76.5 ± 9.0	377 ± 19	1.5 ± 0.6	1.1 ± 0.1	2.3 ± 0.9	3.9 ± 1.8 (is)	0.4 ± 0.2 (me)
*Hevea spruceana*	i	nf	60-100	1000-1740	1557 ± 160.4	36.0 ± 2.0	36.1 ± 0.7	28.3 ± 5.0	347.0 ± 9.4	7.3 ± 1.4	22.4 ± 2.8	16.7 ± 3.7	54.6 ± 23.2 (m)	0.6 ± 0.5 (a)
			7-17	800-1000	909 ± 42.4	34.8 ± 1.9	35.5 ± 0.8	28.1 ± 4.5	350.7 ± 6.6	7.2 ± 2.2	21.3 ± 5.3	16.5 ± 4.4	39.6 ± 5.6 (m)	0.6 ± 0.3 (a)
			11-20	300-800	557 ± 133	31.9 ± 2.6	31.8 ± 2.0	63.9 ± 16.6	358.9 ± 11.6	6.8 ± 1.7	14.7 ± 4.8	31.6 ± 14.1	91.7 ± 75.0 (m)	0.7 ± 1.3 (a)
			237-428	582-749	603 ± 25.0	28.6 ± 2.2	27.9 ± 2.3	85.5 ± 10.7	383 ± 18	3.5 ± 1.2	1.0 ± 0.5	4.1 ± 2.1	9.0 ± 2.8 (m)	0.9 ± 0.6 (a)
*Pseudobombax munguba*	v	nf	49-84	1000-1727	1453.2 ± 191.5	34.1 ± 1.2	34.0 ± 1.3	37.3 ± 10.9	341.0 ± 9	4.4 ± 1.1	2.4 ± 0.7	2.1 ± 0.6	-	13.2 ± 6.1 (me)
			6-12	800-1000	913.3 ± 62.0	32.1 ± 1.0	31.8 ± 1.4	54.2 ± 14.5	347.4 ± 6	4.9 ± 2.1	1.7 ± 0.4	2.4 ± 0.6	-	10.0 ± 6.1 (me)
			13-30	300-800	533.9 ± 161.0	30.2 ± 1.8	30.3 ± 1.8	65.3 ± 13.8	352 ± 9	5.1 ± 1.2	1.4 ± 0.5	3.2 ± 0.9	-	14.3 ± 3.0 (me)
		f	277-490	582-837	603 ± 25.0	28.6 ± 2.2	27.9 ± 2.3	85.5 ± 10.7	383 ± 18	7.2 ± 1.0	2.4 ± 0.7	8.5 ± 4.7	-	6.3 ± 1.9 (me)
*Hura crepitans*	v	nf	23-52	1000-1656	1244 ± 188.9	33.4 ± 1.0	34.5 ± 0.7	20.4 ± 22.0	164.1 ± 172	4.5 ± 4.8	1.4 ± 1.5	1.9 ± 2.5	-	8.5 ± 4.4 (me)
			10-25	800-1000	909.1 ± 55.5	33.2 ± 1.0	34.3 ± 0.9	24.6 ± 22.8	189.8 ± 170	5.1 ± 4.6	1.6 ± 1.5	2.2 ± 2.4	-	8.6 ± 5.2 (me)
			45-89	300-800	562.8 ± 140.7	31.7 ± 0.8	32.8 ± 1.0	44.3 ± 20.8	287.5 ± 127.6	7.0 ± 3.3	2.0 ± 1.0	3.2 ± 2.1	-	16.8 ± 12 (me)
			244-432	340-463	620 ± 40.5	32.0 ± 2.1	32.0 ± 2.4	70.0 ± 11.9	368 ± 20	5.5 ± 0.9	2.5 ± 1.0	1.2 ± 0.9	-	3.4 ± 1.2 (me)
*Pouteria glomerata*	v	nf	221	562-590	573.1 ± 7.7	32.0 ± 1.4	31.7 ± 1.7	65.4 ± 14.5	349 ± 25	4.7 ± 1.4	9.4 ± 5.5	17.6 ± 10.5	-	-
		f	345-363	583-846	636.2 ± 77.5	34.8 ± 3.1	34.9 ± 3.1	55.4 ± 14.5	357 ± 15	3.0 ± 1.0	2.3 ± 0.8	3.9 ± 3.3	-	-

Given are day time averages (n = individual measurement data; 5 minutes averages each) over one nonflooded (nf) day and one flooded (f) day after two months and three weeks of inundation of three tree individuals ± SD. Várzea (v), igapó (i). En = Environment.

On the other hand, slight differences in leaf temperatures might have affected the physiology of the plant in some cases (see Table 2**2**). Leaf temperatures were recorded at 2–6 °C above the reported temperature optima for tropical trees (25–30 °C, (Larcher [2003])). Leaf temperatures of *Garcinia macrophylla* and *Hevea spruceana* were lower during the experiments under flooded conditions than before inundation, in both cases approaching the temperature optimum reported by Larcher ([2003]). Similarly, leaf temperatures of *Hura crepitansPouteria glomerata* and *Pseudobombax munguba* did not differ very much. Thus critical temperature effects on physiology and VOC emissions could be excluded. It is noteworthy to mention, that as in the short-term flooding experiment, enclosure temperature was similar to leaf temperature. Plants were maintained under natural CO_2_ conditions, with ambient CO_2_ concentrations of 342–382 ppm. Ambient relative humidity was 19.2 - 52% lower for the nonflooded experimental series, except for the days when *Pouteria glomerata* was investigated.

#### Soil conditions

Due to the low solubility of O_2_ in water and its consumption by respiring roots and microorganisms, the soil of inundated plants becomes hypoxic (Visser *et al.*[2003]). In this study, the oxygen concentrations in the water measured at surface level reached values between 4.3 to 7.2 mg l^-1^ corresponding to O_2_ conditions occurring naturally in Amazon water (Furch and Junk [1997]). No difference between long-term and short-term inundation was found.

### Morphological adaptation

In oxygen depleted soil, plants have evolved a wide range of characteristic responses that appear to reduce the impact of the stress (Parolin *et al.*[2004]). Accordingly, adventitious roots in the oxygenated layer at the surface of the water table, as well as lenticels at the stem above the water table were observed in our studies. Both features improve the plant’s oxygen status by facilitating the entry of oxygen into the root and the stem. These morphological formations were observed in all plants during the long-term flooding period, but not during the short-term flooding experiment. The pioneer tree *Pseudobombax munguba* showed the longest and most developed adventitious roots and lenticels. None of the flooded tree species had signs of senescence except for *Pouteria glomerata*, and new leaves were still observed on the apical region after the long period of inundation.

### Physiological adaptation and VOC emission responses to waterlogging conditions

#### Physiology and VOC emissions during short-term flooding

**Figure 1:**
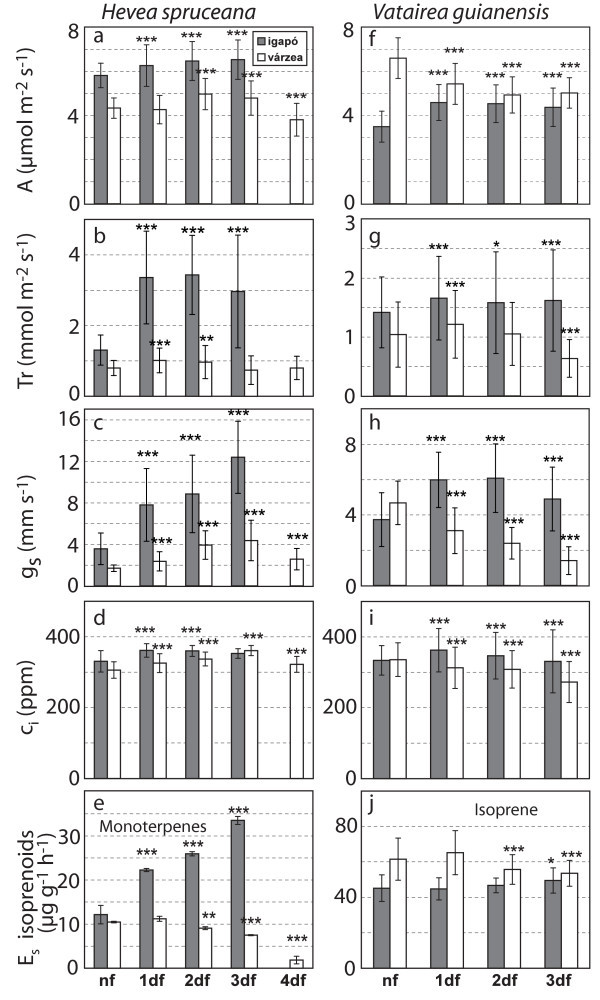
**Short-term flooding: Mean values averaging over 5 minutes during day time (Day time averages) for Assimilation (A) in μmol m**^**-2**^ **s**^**-1**^**, transpiration (Tr) in mmol m**^**-2**^ **s**^**-1**^**, stomatal conductance (gs) in mm s**^**-1**^**, leaf internal CO**_**2**_**concentration (Ci; ppm) with their standard deviations (SD) and Standard emissions (E**_**s**_**) of monoterpenes and isoprene.** Data are derived from two individuals each of *Hevea spruceana* (n = 142 per day) and *Vatairea guianensis* (n =142 per day) from várzea (light grey) and igapó (dark grey) given on a dry weight basis in μg g^-1^ h^-1^ ± SD. The significances of the differences between nonflooded (nf) and 1, 2, 3 and 4 days flooded (df) were tested with ANOVA and the Tukey Test; * = P-value < 0.01 F ratio is significant, ** =,P-value < 0.001 F ratio is very significant. *** = P-value < 0.0001 F ratio is highly significant.

**Figure 2:**
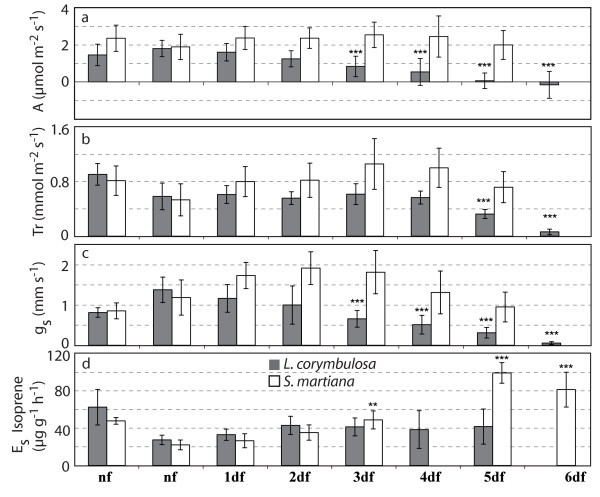
**Short-term flooding: Mean values averaging over 5 minutes during day time (Day time averages) for assimilation (A) in μmol m**^**-2**^ **s**^**-1**^**, transpiration (Tr) in mmol m**^**-2**^ **s**^**-1**^**, stomatal conductance (gs) in mm s**^**-1**^ **± SD and Standard Emissions of isoprene in μg g**^**-1**^ **h**^**-1**^ **± SD.** Data are derived from one individual each of *Laetia corymbulosa* (dark grey) (n =74-144 per day) and *Salix martiana* (light grey) (n = 30-144 per day) from várzea with average light conditions during the experiment of 185 ± 36 and 216 ± 39 μmol m^-2^ s^-1^ for *L. corymbulosa* and *S. martiana*, respectively. The significance of the differences between nonflooded (nf) and 1, 2, 3 and 4 days flooded (df) were tested with ANOVA and Tukey Test; * = P-value < 0.01 F ratio is significant, ** =,P-value < 0.001 F ratio is very significant, *** = P-value < 0.0001 F ratio is highly significant. These data were derived from an unpublished data set obtained with these Amazonian tree species in the year 2000 at the University of Oldenburg. Data upon the release of the fermentation products gained within these studies were published elsewhere (Rottenberger *et al.*[2008]).

The physiological response to flooding was very variable among the different plant species studied and also between the várzea and igapó environments (see Figure 1a-j). Rates of assimilation and transpiration, stomatal conductance and the internal concentration of CO_2_ were found to be significantly higher (P<0.0001) after only one day of flooding in the case of the igapó species *Hevea spruceana* and *Vatairea guianensis*. This increase of assimilation and stomatal conductance was further enhanced in the case of *Hevea spruceana* during the following flooding days. *Hevea spruceana* from várzea reacted less pronounced, whereas *Vatairea guianensis* from várzea showed a significant reduction in its physiological parameters from the first day of inundation (P < 0.0001). The common várzea species *Laetia corymbulosa* exhibited a more or less constant decrease of assimilation, stomatal conductance and transpiration (Figure 2a-c). In contrast, the physiology of the várzea pioneer tree *Salix martiana*, that is often completely flooded under natural conditions, was briefly affected by root inundation (Figure 2a-c), showing an increase of assimilation, transpiration and stomatal conductance in the first days of inundation.

Isoprenoid emissions can be affected by periods of short-term flooding in different manners depending on the tree species, the origin and the isoprenoid species. For example, the change of monoterpene emission for the tree species *Hevea spruceana* was larger than the isoprene emission by *Vatairea guianensis* (see Figure 1e and j). *Hevea spruceana* from igapó showed an increase of monoterpene standard emission factors (E_s_) whereas the várzea species showed a decrease (see Figure 1e). *Vatairea guianensis* from várzea showed a decline of isoprene emissions whereas no effects were observed in the case of the igapó species (see Figure 1j). On the other hand isoprene emission by *Laetia corymbulosa* from várzea was not affected by flooding. *Salix martiana* from várzea showed higher isoprene emission during the last days of inundation (see Figure 2d).

**Figure 3:**
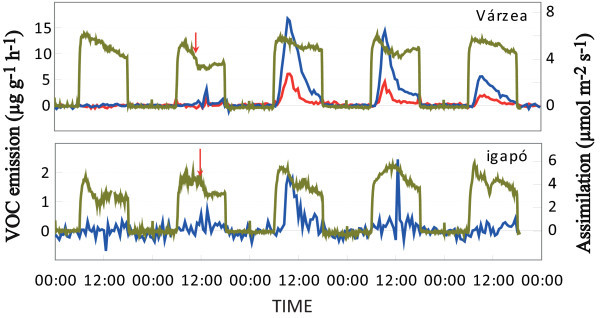
**Assimilation (A) (green line) in μmol m**^**-2**^ **s**^**-1**^**, and ethanol (blue line) and acetaldehyde (red line) emissions in μg g**^**-1**^ **h**^**-1**^**during the short-term flooding experiment on*Vatairea guianensis*from várzea and igapó.** The red arrow represents the moment of inundation.

The emission of ethanol and/or acetaldehyde was observed during the short-term inundation periods (3 days) with the tree species *Vatairea guianensis* (see Figure 3) exhibiting some differences depending on the origin, i.e. várzea or igapó regions. Igapó species seemed to adapt more quickly to the stress produced by flooding, emitting only small amounts of ethanol on the first day after the inundation and even less on the second day. The várzea species emitted higher quantities of ethanol plus acetaldehyde from the first day of inundation. The emission of both acetaldehyde and ethanol decreased during the three days of measurement. *Hevea spruceana* did not release any ethanol or acetaldehyde.

#### Physiology and VOC emissions after long-term flooding

**Figure 4:**
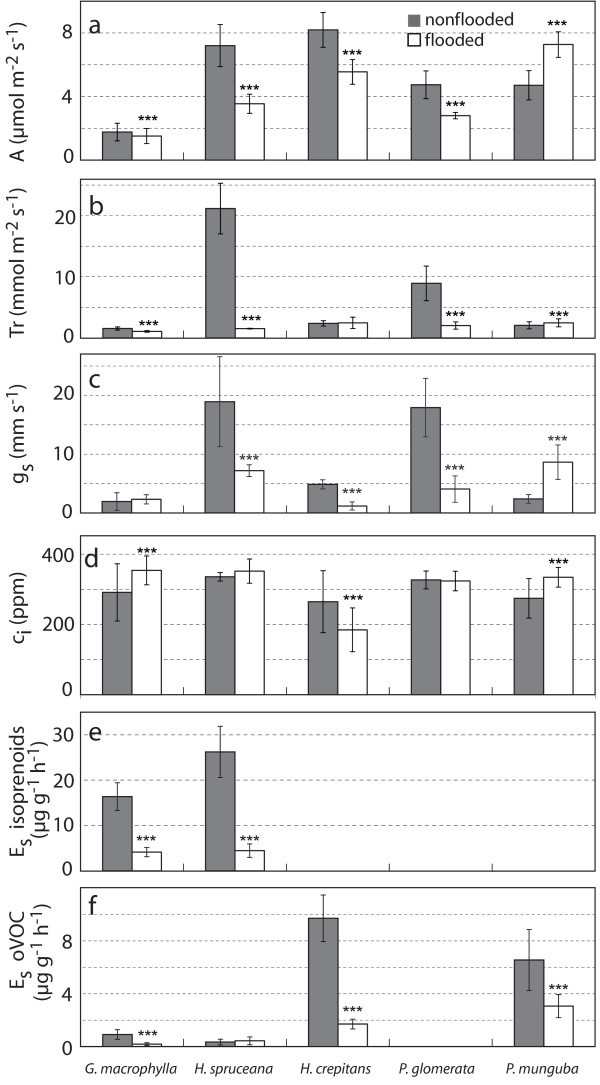
**a-f Mean values averaging over 5 minutes during day time (Daytime averages) for assimilation (A) in μmol m**^**-2**^ **s**^**-1**^**, transpiration (Tr) in mmol m**^**-2**^ **s**^**-1**^**, stomatal conductance (gs) in mm s**^**-1**^**and internal CO**_**2**_**concentration (Ci) in ppm under maximal photon flux density for three replicates of*Garcinia macrophylla, Hevea spruceana, Hura crepitans, Pouteria glomerata*and*Pseudobombax munguba*under nonflooded conditions (n = 66–142 per day) ± standard deviation (SD) and after two months and three weeks inundation (n = 142 per day) ± standard deviation (SD).** Standard emission factors (E_s_) for isoprene in the case of *Garcinia macrophylla* from igapó, for monoterpenes in the case of *Hevea spruceana* from igapó, for methanol in the case of *Garcinia macrophylla, Hura crepitans* and *Pseudobombax munguba* and for acetone in the case of *Hevea spruceana* are given in μg g^-1^ h^-1^ under nonflooded conditions (n = 142 per day) and after two months and three weeks inundation (n = 142 per day) ± SD. Differences between nonflooded (grey) and flooded conditions (white) were tested with ANOVA and the Tukey Test. When the P-value < 0.01 the F ratio is significant (*), when the P-value < 0.001 the F ratio is very significant (**) and when the P-value < 0.0001 the F ratio is highly significant (***).

Under waterlogging conditions, assimilation decreased significantly (P < 0.0001) in all species investigated except for *Garcinia macrophylla* which did not show any significant difference. *Pseudobombax munguba* showed higher photosynthetic rates after the long-term flooding period (Figure 4a). Photosynthesis correlated well with the pattern of stomatal conductance. Assimilation changes may be related to changes of stomatal conductance, transpiration, and internal CO_2_ concentration (C_i_) with substantial variability depending on the plant species as shown in Figure 4. However, two species, *Garcinia macrophylla* and *Pseudobombax munguba,* demonstrated a remarkable stability. They were obviously well adapted to long-term flooding exhibiting no changes or increased assimilation with increased conductance and C_i_.

Isoprenoid emission was clearly affected by long-term waterlogging. Emission factors of isoprene and monoterpenes determined for the isoprene emitter *Garcinia macrophylla* and the monoterpene emitter *Hevea spruceana* dropped by ~75% and ~83%, respectively (see Figure 4). There is a general tendency of down-regulation of the physiological parameters and VOC emissions in most of the plant species investigated after a flooding period of around 3 months. This down regulation was not observed in the case of the physiological parameters of *Garcinia macrophylla* and *Pseudobombax munguba,* where a maintenance or up regulation was observed (see Figure 4).

Similarly, other VOC emissions such as methanol emissions by *Garcinia macrophylla, Hura crepitans* and *Pseudobombax munguba*, showed a decrease of 53–82% of the standard emission factors after the long-term flooding. In addition to methanol release, a low acetone emission in the range of 0.34 - 0.44 μg g^-1^ h^-1^ was observed for *Hevea spruceana*, but no flooding related effects could be detected.

## Discussion

Investigations of VOC emission and assimilation by several Amazonian tree species from two floodplain ecosystems incubated under short-term and long-term inundation demonstrated non uniform responses.

### Short-term flooding

Igapó and várzea seedlings of the monoterpene emitter *Hevea spruceana* exhibited a counter directional behavior with a strong increase as found for the igapó and a decrease in case of the várzea species. In case of the isoprene emitting species *Vatairea guianensis* the várzea species generally showed a higher emission than the igapó species but both did not exhibit strong responses to inundation (Figure 1**1**). The isoprene emitters *Laetia corymbulosa* and *Salix martiana* did not show significant emission changes during the first 4 days of inundation. However, it is interesting to note that the pioneer tree *Salix martiana* exhibited a strong significant increase over the last two days with still stable assimilation rates whereas *Laetia*, though with continuing isoprene emissions, did not exhibit physiological stability.

Isoprenoid emission can be affected by biotic and abiotic stress (Beauchamp *et al.*[2005], Davison *et al.*[2008], Ibrahim *et al.*[2008], Plaza *et al.*[2005], Sharkey *et al.*[2008]) in close relationship to photosynthesis (Delwiche and Sharkey [1993], Ferrieri *et al.*[2005], Kuhn *et al.*[2002], Schnitzler *et al.*[2004]). Thus, under stress situations like flooding, a higher carbon loss by VOC emission in relation to photosynthesis might be expected (Kesselmeier *et al.*[2002]), especially in view of other compounds other than isoprenoids (Holzinger *et al.*[2000], Rottenberger *et al.*[2008]). Such an enhancement of VOC emissions could be confirmed in this study for *Hevea spruceana* from igapó and *Salix martiana* from várzea, with a remarkable increase of isoprenoid release. An increase of assimilation as observed in the case of the igapó species may balance the carbon loss. In contrast, a decrease of VOC emission is observed for *Hevea spruceana* and *Vatairea guianensis* from várzea, suggesting a decrease of this secondary metabolism in order to preserve the primary metabolism. On the other hand, *Laetia corymbulosa* from várzea maintained isoprene emission during the flooding period although its assimilation decreased.

Leaves of plants subjected to root waterlogging conditions can emit other VOCs such as ethanol and acetaldehyde in response to root anoxia (Holzinger *et al.*[2000], Kennedy *et al.*[1992], Kreuzwieser *et al.*[1999b], MacDonald and Kimmerer [1993], MacDonald *et al.*[1989], Rottenberger *et al.*[2008], Schlüter *et al.*[1993], Visser *et al.*[2003]). The ethanol is produced in the roots due to alcoholic fermentation under anoxia, a strategy for gaining energy under an insufficient oxygen supply in the roots. However, its accumulation in the roots can be toxic (Kennedy *et al.*[1992]). Therefore it is transported to the leaves via the transpiration stream, where it can be directly emitted to the atmosphere, or oxidized to acetaldehyde and/or acetate, both of which also partly escape into the atmosphere. Acetate can be converted to acetyl-CoA by acetyl-CoA synthetase and then reenter the metabolism pathways such as the TCA cycle or lipid synthesis (MacDonald and Kimmerer [1993]). This may have been the case for *Hevea spruceana* from both environments, where no ethanol or acetaldehyde emissions were detected suggesting a strategy for economizing energy by re-metabolization such oxygenated VOCs. This is in close accordance with the low ethanol emissions found by the well adapted *Salix martiana* (Rottenberger *et al.*[2008]). On the other hand, *Vatairea guianensis* from both environments seemed to be the worst adapted to flooding conditions, since emissions of oxygenated VOCs were detected from the first day of inundation and decreased continuously with time. Nevertheless, *Vatairea guianensis* from igapó could cope a little bit better with the anoxia, emitting only ethanol on the first and second day of inundation and in lower quantities than emitted from *Vatairea guianensis* from várzea. However, the várzea species emitted acetaldehyde in addition until the third day of inundation, suggesting a high ADH (Alcohol Dehydrogenase) activity (Parolin *et al.*[2004]), as already observed in case of *Laetia corymbulosa* in previous studies performed by Rottenberger *et al.* ([2008]).

### Long-term flooding

The long-term flooding experiment demonstrated the potential capacity to produce morphological adaptations forming adventitious roots and hypertrophy of lenticels. These adaptations may improve the internal oxygen status by facilitating the entry of oxygen into the root and the stem. Such modifications have been observed in juvenile and adult individuals for several species (Parolin [2001a]) and lead to the question of how the emission of VOC changes under long-term root anoxia. A 3 month period of inundation may not be a long period of inundation for these plants, if we take into account that the investigated plants can be subjected to longer inundation periods in nature with roots below the water table for years (Wittmann *et al.* ([2004, 2010b]) or totally flooded up to 210 days year^-1^ (Junk [1989]). To our knowledge no such measurements have been performed to date. The studies, as reported here, are a first step in investigating long-term behavior and should be regarded as preliminary. In three of the plant species measured, assimilation rates were lower under waterlogging conditions after long-term inundation, confirming the results already reported by Parolin *et al.* (Parolin [2000a]). In contrast, the pioneer tree *Pseudobombax munguba* exhibited a higher assimilation rate under inundation as compared to non-flooding conditions. Well-adapted behavior could also be observed for the pioneer tree *Cecropia latiloba*, with a mean CO_2_ uptake which did not significantly differ from the non-inundated period, though in special cases increased assimilation could be observed (Parolin [2000a]). Also *Garcinia macrophylla* tolerated long-term flooding conditions, exhibiting no physiological changes.

This study reports for the first time the effects of long-term flooding on emissions of isoprenoids and oxygenated VOC, such as methanol and acetone. Substantial decreases (75-83%) of isoprene and monoterpene emissions were observed in the case of *Garcinia macrophylla* and *Hevea spruceana*, whereas CO_2_ assimilation rates were found to be variable with a reduction of 14% (*Garcinia macrophylla*) and 51% (*Hevea spruceana* - várzea). Reduction of methanol emission was also observed. This latter tendency might be the result of growth reduction due to the stress conditions (Parolin and Ferreira, [1998]), or in accordance with a developmental stage (Hüve *et al.*[2007]). The low, but detectable, acetone emissions as found for *Hevea spruceana* were not affected.

The lack of ethanol and acetaldehyde emissions under long-term flooding conditions is of high interest for biosphere atmosphere exchange under natural conditions. Based on these limited investigations, we may assume that this metabolic strategy is relevant only for the first days of adaptation to a lack of oxygen in the roots. Under long-term flooding periods the alcoholic fermentation seems to lose its role in the gain of energy. The accumulation of organic acids and aerobic respiration which is dependent on oxygen transport, begin to play a more dominant role at this stage as is observed in the tropical tree *Astrocaryum jauari* by Schlüter *et al.* ([1993]). Under these conditions, the respiration process is obviously fed by a high consumption of reserve materials under low oxygen partial pressure (Schlüter *et al.*[1993]).

Finally, beside taxonomic issues, the time of colonization and adaptation of floodplain trees to the inundation area may play an important role. According to Kubitzki ([1989]), trees have migrated from the adjacent uplands, colonizing the river floodplains. Therefore, differences of flooding tolerance between the plant species and the related metabolic balance could represent differences in the time of colonization. Plant species better adapted may have colonized the inundation areas earlier, as suggested by Piedade *et al.* ([2000]), Parolin *et al.* ([2010a]) [b] and Wittmann *et al.* (2010b)Wittmann and De Oliveira [(2010a)].

## Conclusions

The effects of root anoxia on VOC emissions reported so far were based on investigations of tree seedlings under controlled or semi-controlled conditions. Response to short-term anoxia is highly variable and related to species and ecotype. The release of fermentation products can be regarded as a sign of an ecophysiological advantage to gain energy under root anoxia caused by waterlogging. Physiological activity as indicated by ethanol accumulation in the roots in course of a functional respirational capability can be observed even after 200 days of inundation (Parolin, [2009]). Consequently, a release of the toxic metabolic compound ethanol-potentially through the leaves after transport with the transpiration stream - might occur. But within our experiments we found such an emission only after short term flooding conditions. It is not clear whether this is due to a missing ethanol accumulation, missing transport or recovery of the ethanol and its metabolic products under long term conditions. A general decrease of physiological activity is however reflected by the decrease of photosynthesis and an even stronger decrease of isoprenoid emissions under our experimental long term inundation. Decrease of isoprenoid emissions may be regarded as an adaptation of the plants by decreasing the VOC carbon loss under these special stress conditions. The results indicate that there is a gap in our knowledge which needs to be better understood. Nothing is known about the behavior and VOC emissions of adult trees under field conditions which are adapted to long-term flooding being characteristic for Amazonian floodplains. Field studies under such conditions are challenging, but should be intensified to interpret and understand ecophysiological advantages and to clarify the influence of regular flooding of large Amazonian areas on ecophysiology and VOC emission.

## Methods

### Plant material

Seedlings of 8 widely distributed tree species from the Central Amazonian floodplain areas were chosen for this study (see Table 3). *Vatairea guianensis* Aubl. (Fabaceae), *Hevea spruceana* (Benth.) Müll. Arg*.* (Euphorbiaceae) and *Garcinia macrophylla* (Mart.) Planch. & Triana (Clusiaceae) are of commercial importance and occur in both the várzea and igapó floodplain areas (Ferreira [1997], Parolin [2000b], Wittmann *et al.*[2006], Worbes [1986]). *Hevea spruceana* and *Garcina macrophylla* are common also in terra firme forest, but not *Vatairea guianensis* (Wittmann *et al.*[2006]). For our experiment, *Vatairea guianensis* and *Hevea spruceana* were collected from várzea and igapó, respectively, and *Garcinia macrophylla* from igapó. Species like the pioneer tree *Pseudobombax munguba* (Mart. & Zucc.) Dugand (Bombacaceae) and two species commonly used in the wood industry namely *Hura crepitans* L. (Euphorbiaceae) and *Pouteria glomerata* (Miq.) Radlk. (Sapotaceae) *-* were also collected for our experiments as they are widely distributed in the várzea environment.

**Table 3:** **Plant species, family, ecosystem, functional type, occurrence and measured specific leaf weights (SLW) of the 8 tropical plant species investigated from várzea (v) and igapó (i)**

Plant species	SLW [g m^-2^]	Family	Ecosystem^1^	Functional Type^2^	Countries of occurence^3^
***Garcinia macrophylla***	142 (i)”	Clusiaceae	a, b*, c, d, i	evergreen	Bolivia, Brazil, Ecuador, French Guiana, Guyana, Peru, Suriname, United States, South-East Asia
(Mart.) Planch. & Triana					
***Hevea spruceana***	24 (v)’27 (i)’43 (i)”	Euphorbiaceae	a*, b*, d	deciduous/brevi -deciduous	Bolivia, Brazil, Colombia, Costa Rica, Peru
(Benth.) Müll.Arg.					
***Hura crepitans***L.	45 (v)”	Euphorbiaceae	a*, c, i	brevi	Netherlands Antilles, Benin, Bolivia, Brazil, Bahamas, Belize, Central African Republic, Cote d’Ivoire, Colombia, Costa Rica, Cuba, Domincan Republic, Ecuador, French Guiana, Guatemala, Indonesia, Lao Peoples Democratic Republic, Madagascar, Martinique, Mexico, Nicaragua, Panama, Suriname, El Salvador, Togo, Thailand, Trinidad and Tobago, Chinese Taipei, Tanzania, United States, Venezuela, Vietnam
				-deciduous	
***Laetia corymbulosa***	58 (v)’	Flacourtiaceae	a*	brevi-deciduous	Bolovia, Brazil, Colombia, Ecuador, Perú
Spruce ex Benth.					
***Pouteria glomerata***	68 (v)”	Sapotaceae	a*, c, d, e, f, g, h	evergreen	Argentina, Bolivia, Brazil, Colombia, Costa Rica, Ecuador, French Guiana, Guatemala, Guyana, Honduras, Mexico, Panama, Peru, Paraguay, Suriname, El Salvador, United States, Venezuela
(Miq.) Radlk.					
***Pseudobombax munguba***	65 (v)”	Malvaceae	a*, b, d	deciduous	Brazil, Colombia, Ecuador, Peru
(Mart. & Zucc.) Dugand					
***Salix martiana*** (Leyb)	23 (v)’	Salicaceae	a*	evergreen	Peru, Brazil
***Vatairea guianensis*** Aubl.	36 (i)’26 (v)’	Fabaceae	a*, b*, c, d, e	deciduous	Brazil, Colombia, French, Guiana, Guyana, Peru, Venezuela

1 Missouri Bot Garden, New Bot. Garden, Royal Bot. Gardens Kew, INPA-Herbarium and Wittmann pers. com.

2 *Schöngart et al.,*[2002].

3 Global Biodiversity Informations Facility: http://data.gbif.org* Indicates the plant’s environment selected for the measurement.

a) Várzea of Central Amazonian.

b) Igapó of Central Amazonian.

c) Amazonian Terra Firme.

d) Orinoco basin.

e) Atlantic rainforest (nonflooded).

f) Brazilian Pantanal (nonflooded).

g) Brazilian Pantanal (flooded).

h) Cerrado.

i) Central America.

(i) igapó.

(v) várzea.

’ short-term flooding experiment.

” long-term flooding experiment.

Seedlings of the várzea were collected at the bank of the Ilha da Marchantaria (03°15´S, 59°58´W), an island located in the Solimões River. Igapó species were collected at the bank of the Tarumã Mirim (03°08´S, 60°01´W) an affluent of the Rio Negro. *Hevea spruceana* and *Vatairea guianensis* occurring in várzea and igapó as well were collected to compare the influence of the two different origins. Seedlings were potted in soil collected at the original habitat of the plant. Measurements were started at the earliest after one month of acclimatization. Plants were kept under natural light conditions, protected from insects with mosquito nets and were irrigated daily with water from an artesian well, at the INPA, Manaus, Brazil. Mean temperature conditions of 30 °C and relative humidity values of 78–91% were recorded. Ambient CO_2_ concentrations were in the range of 335–408 ppm.

In addition we performed an analysis of unpublished data of flooding effects on the isoprenoid emission of *Laetia corymbulosa* Spruce ex. Benth. (Flacourtiaceae) and *Salix martiana* Leyb. (formally called *S. humboldtiana* var. *martiana* (Leyb.) Anders) (Salicaceae) as obtained in one of our recent studies (Rottenberger *et al.*[2008]). *Laetia corymbulosa* is one of the most abundant species from the low várzea areas (Parolin [2002], Wittmann *et al.*[2006]). *Salix martiana* is a fast growing and light demanding pioneer species occurring mainly in the low elevation sites of the várzea forest (De Simone *et al.*[2003b], Parolin [2000b]). Both tree species are evergreen and well adapted to flooding conditions (De Simone *et al.*[2003a], Schöngart *et al.*[2002]).

### Gas exchange measurements with enclosed plants

#### Enclosures

All measurements were performed with a dynamic enclosure system consisting of two Teflon-film enclosures, one enclosing the plant and one empty reference enclosure, as described in detail earlier (Bracho-Nunez *et al.*[2011], Dindorf *et al.*[2006,]Kesselmeier *et al.*[1993, 1996, 1997], Kuhn *et al.*[2000][2002], Schäfer *et al.*[1992]). The enclosures were set up in the surroundings of the Max Planck station at the Instituto Nacional da Pesquisa da Amazônia (INPA), Manaus, Brazil. All experiments were performed outside the building in a semi-natural environment of the INPA campus with a remnant terra firme forest. Depending on the plant size, a small (9 l) or a big enclosure (100 l) was chosen. The air was pumped (Teflon membrane pumps, Vacuubrand, Germany) through an ozone scrubber (a filter of copper nets covered with MnO_2_, Ansyco, Germany) to avoid oxidant interferences with the VOC measurements. The air flow chosen allowed for an exchange of the total enclosure volume between 1 and 5 minutes. Branches, or total above ground plant parts were enclosed and measured. All tubes connecting the enclosure with instruments were heated to a temperature above ambient temperature (~45 °C) in order to avoid condensation of volatiles (Larsen *et al.*[1997]). The mixing ratio, E_s_, of each compound was calculated according to Equation (1) taking into account the measured concentration difference between the reference (empty) and the sample (with the plant) enclosure (*Δc* = *c*_*sample*_*- c*_*reference*_) in [nmol mol^-1^, the enclosure flush rate *Q* in [l h^-1^, and the leaf dry weight (dw) in [g] and the molecular mass M in [g l^-1^. Leaf dry weight was determined at the end of the experiment and extrapolated to the earlier measurements.1

Measurements were performed under semi-controlled conditions and ambient relative humidity and temperature. Relative humidity and temperature from each enclosure was monitored by the use of two combined temperature/humidity sensors (Model Rotronics MP-100A, Walz, Germany). In most cases additional photosynthetic active radiation (PAR) was constantly provided by a LED system consisting of four double chains of a mixture of red, blue, and white light constructed and designed by the electronics department of the MPI for Chemistry in Mainz, Germany. This LED system was placed perpendicular to the enclosure and the gaps between the LED groups were closed by a reflecting film in order to obtain a homogenous distribution of the light in the enclosure. PAR was measured with a quantum sensor (Model SB 190, Licor, USA) inside the enclosure at different heights before and after the measurements. During the long-term inundation experiment the control of light intensities was not possible during nonflooded measurements. Therefore natural sun light was used. One minute averages for enclosure and leaf temperatures were measured with thermocouples (Type E, Chrom-Constantan, OMEGA) and were recorded with a datalogger CR23X (Campbell Scientific Ltd. Shepsherd, UK). All other micrometeorological and physiological parameters were recorded simultaneously by a V25control unit, built at the MPI for Chemistry, on the same time scale.

#### Assimilation/transpiration measurements

The physiological parameters photosynthesis and transpiration were measured with a CO_2_/H_2_O infrared gas analyzer (LI-COR Inc. 7000, Lincoln, Nebraska, USA). This equipment was operated in differential mode and received the absolute reference concentration signal from a second CO_2_/H_2_O infrared gas analyzer (LI-COR Inc. 7000, Lincoln, Nebraska, USA). Nitrogen (N_2_ 5.0, Messer Griesheim, Germany) was used as the reference gas for the absolute CO_2_ and H_2_O in the reference enclosure. The analyzer was calibrated prior to the experiments by using a CO_2_ calibration gas standard (512 ± 2 ppm CO_2_ in synthetic air, LI-COR, Lincoln, Nebraska, USA), and a dew point generator for the calibration of water vapor (Li 610; LI-COR, Lincoln, Nebraska, USA). At the end of each experiment the calibration of the analyzer was checked and the signal response was corrected for sensitivity and zero drifts as a function of time. The signal response of the instrument was also corrected for temperature effects and with regard to the offset of specified and measured reference concentrations. Stomatal conductance was calculated according to Pearcy, Schulze & Zimmermann (Pearcy *et al.*[1989]).

In addition measurements of CO_2_ exchange and transpiration for individual leaves for all tree species were made using a GFS3000 Portable Gas Exchange System (Walz, Germany) in order to determine the light saturation of photosynthetic uptake of CO_2_ for the purpose of characterizing the adaptation of all tree species to the variable environment at the surroundings of the INPA-Max Planck Project at the Instituto Nacional da Pesquisa da Amazônia (INPA), Manaus.

#### VOC determination

VOCs were analyzed with the online Proton Transfer Reaction - Mass Spectrometer (PTR-MS) which was connected to the enclosures by 1/8 inch tubing. The PTR-MS technique has been thoroughly reviewed elsewhere (Blake *et al.*[2009], Lindinger *et al.*[1998a, b]). The instrument was maintained in an air conditioned room during all experiments. The sample and reference enclosure and the background signal were probed alternately. The instrument was operated in selected ion-monitoring mode at standard operation conditions (E/N = 130 Td; E electric field strength, N buffer gas number density, 1 Td = 10^-17^ cm² V molecule^-1^) at a drift voltage of 600 V. The considerable background signal, probably caused by desorption of impurities inside the sampling system and the drift tube (Steinbacher *et al.*[2004]), was determined by applying VOC free air generated by a catalytic converter (Zero-air generator, Parker Co., USA) to the instrument. This offset was subtracted from the sample and reference signal and the emission rate was calculated according to Eq.1. Emission rates calculated on a leaf dry weight basis in μg g^-1^ h^-1^ were standardized using a mathematical algorithm developed by Guenther *et al.* ([1993, 1995]) and Guenther ([1997]) in following referred to as G93. This algorithm was developed to predict isoprene emissions, but predicts monoterpene emissions very well too (Ciccioli *et al.*[1997], Kesselmeier *et al.*[1997], Kuhn *et al.*[2002][2004]). G93 describes the emission of volatile organics as a function of a basic emission strength (i.e. a standard emission factor or basal emission rate, E_s_) related to the environmental parameters photosynthetic active radiation (PAR, 1000 μmol m^-2^ s^-1^) and temperature (30° C).

The main emissions detected by PTR-MS were methanol (m33), acetaldehyde (m45), ethanol (m47), acetone (m59), isoprene (m69) and monoterpenes (m137, fragment on m81). It is important to note that different types of monoterpenes cannot be distinguished separately with PTR-MS. Therefore, standard emissions factors calculated for monoterpenes with the PTR- MS in this study always refer to the sum of all monoterpenes. The PTR-MS instrument was calibrated using a gas standard (Apel Riemer, USA or Deuste Steininger GmbH, Germany) containing most, except ethanol, of the given target VOCs in nitrogen (± 5% accuracy for Apel Riemer and ± 10% accuracy for Deuste standard) and diluted with synthetic air to concentrations of 0.5 - 10 ppb. The volume mixing ratio of ethanol was calculated using simple ion-molecule reaction kinetics as reported elsewhere (Hansel *et al.*[1995], Wisthaler *et al.*[2001]). The detection limit of the PTR-MS was estimated as being 1.96 times the standard deviation of the empty enclosures concentrations (at the 95% confidence level) and was typically 1.153 ppb for methanol, 291 ppt for acetone, 445 ppt for isoprene and 574 ppt for monoterpenes.

### Experimental schedule

All the measurements were performed under semi-controlled conditions over a two year period, at the Instituto Nacional de Pesquisas da Amazônia **-** INPA botanical garden in Manaus, Brazil with tree seedlings. The investigations were complemented with an evaluation of previous, but unpublished data sets on isoprenoid release carried out with Amazonian tree species in the year 2000 at the University of Oldenburg. In this investigation short-term flooding studies of VOC emissions from *Laetia corymbulosa* and *Salix martiana* were performed also using the PTR-MS technique (Rottenberger *et al.*[2008]). Inundation was achieved by submerging the potted root area. For the short-term experiments, the pots of *Vatairea guianensis* and *Hevea spruceana* from igapó and várzea, respectively, were inundated for three to four days. In accordance with other studies, we regarded this time period to be sufficient for a significant enhancement of VOC emissions (Kreuzwieser *et al.*[1999a], Rottenberger *et al.*[2008]). In view of the limited equipment and time we were able to investigate only two individuals of each species. We regard the results as reliable in terms of emission quality. Quantities may of course be afflicted with larger uncertainties. These experiments took place from the beginning of March to the beginning of May 2007. Plants were enclosed in the evening (around 20:00) and the first data were taken after a minimum time of 10 hours which was deemed to be sufficient for the acclimatization of the plant to the enclosure environment (Dindorf *et al.*[2006], Hayward *et al.*[2004], Llusia *et al.*[2008], Rottenberger *et al.*[2008]). On the second day at noon the plant’s root system was inundated as described above. During the whole experiment measurements of VOC emissions as well as of plant physiological processes were performed online with the PTR-MS and IRGA gas analyzers, respectively. In addition, the O_2_ water concentration was measured for any potential decrease every day at noon (Profiline Dissolved Oxygen Meter Oxi197, WTW, Weilheim, Germany) during the inundation period.

For the long-term experiment we selected three individuals from each of the igapó species *Hevea spruceana* and *Garcinia macrophylla* and the várzea species *Hura crepitans*, *Pouteria glomerata* and *Pseudobombax munguba.* The experiment lasted from July/August to October/November 2006. The measurement schedule was similar to that described above. After the control measurements, the plants were taken out of the enclosures and inundated for two months and three weeks. At the end of this long-term incubation the plants were again enclosed and probed.

The statistical analysis was performed with 5 minutes mean values of physiological as well as emission data und daylight conditions (day time averages). The data obtained after the long term incubation were grouped into light intensity classes in order to get data to be compared with the short time experiment which was performed under lower light intensities due to the differing natural conditions in the wet and dry season. Statistical differences between flooded and nonflooded conditions were performed by one-way ANOVA and proven by the Tukey test. Differences were considered significant at a probability level of P < 0.01 (*), very significant at P < 0.001 (**) and highly significant at P < 0.0001 (***). Two tree individuals (for the short-term experiments) or three (for the long-term experiments) were used for each treatment.

## Abbreviations

C_i_: Internal CO_2_ concentration
INPA: Instituto Nacional da Pesquisa da Amazônia
LBA: Large-Scale Biosphere-Atmosphere Experiment
PAR: Photosynthetic Active Radiation
PTR-MS: Proton Transfer Reaction - Mass Spectrometer
RH: Relative Humidity
T_leaf_: Leaf Temperature
T_enclosure_: Enclosure Temperature
VOC: Volatile Organic Compounds.
